# Common Genetic Determinants of Lung Function, Subclinical Atherosclerosis and Risk of Coronary Artery Disease

**DOI:** 10.1371/journal.pone.0104082

**Published:** 2014-08-05

**Authors:** Maria Sabater-Lleal, Anders Mälarstig, Lasse Folkersen, María Soler Artigas, Damiano Baldassarre, Maryam Kavousi, Peter Almgren, Fabrizio Veglia, Guy Brusselle, Albert Hofman, Gunnar Engström, Oscar H. Franco, Olle Melander, Gabrielle Paulsson-Berne, Hugh Watkins, Per Eriksson, Steve E. Humphries, Elena Tremoli, Ulf de Faire, Martin D. Tobin, Anders Hamsten

**Affiliations:** 1 Atherosclerosis Research Unit, Department of Medicine, Karolinska Institutet, Karolinska University Hospital, Stockholm, Sweden; 2 Pfizer Worldwide Research and Development, Cambridge, United Kingdom; 3 Genetic Epidemiology Group, Department of Health Sciences, University of Leicester, Leicester, United Kingdom; 4 National Institute for Health Research, Leicester Respiratory Biomedical Research Unit, Glenfield Hospital, Leicester, United Kingdom; 5 Dipartimento di Scienze Farmacologiche e Biomolecolari, Università di Milano, Milan, Italy; 6 Centro Cardiologico Monzino, Istituto di Ricovero e Cura a Cattere Scientifico, Milan, Italy; 7 Department of Epidemiology, Erasmus Medical Center - University Medical Center Rotterdam, Rotterdam, the Netherlands; 8 Department of Clinical Sciences, Lunds University, Malmö, Sweden; 9 Department of Internal Medicine, Erasmus Medical Center - University Medical Center Rotterdam, Rotterdam, the Netherlands; 10 Inspectorate for Health Care, The Hague, the Netherlands; 11 Department of Internal Medicine, Skåne University Hospital, Malmö, Sweden; 12 Cardiovascular Research Unit, Department of Medicine, Karolinska Institutet, Karolinska University Hospital, Stockholm, Sweden; 13 Department of Cardiovascular Medicine and the Wellcome Trust Centre for Human Genetics, University of Oxford, Oxford, United Kingdom; 14 Center for Cardiovascular Genetics, Institute of Cardiovascular Science, University College London, London, United Kingdom; 15 Division of Cardiovascular Epidemiology, Institute of Environmental Medicine, Karolinska Institutet, Stockholm, Sweden; Tor Vergata University of Rome, Italy

## Abstract

Chronic obstructive pulmonary disease (COPD) independently associates with an increased risk of coronary artery disease (CAD), but it has not been fully investigated whether this co-morbidity involves shared pathophysiological mechanisms. To identify potential common pathways across the two diseases, we tested all recently published single nucleotide polymorphisms (SNPs) associated with human lung function (spirometry) for association with carotid intima-media thickness (cIMT) in 3,378 subjects with multiple CAD risk factors, and for association with CAD in a case-control study of 5,775 CAD cases and 7,265 controls. SNPs rs2865531, located in the *CFDP1* gene, and rs9978142, located in the *KCNE2* gene, were significantly associated with CAD. In addition, SNP rs9978142 and SNP rs3995090 located in the *HTR4* gene, were associated with average and maximal cIMT measures. Genetic risk scores combining the most robustly spirometry–associated SNPs from the literature were modestly associated with CAD, (odds ratio (OR) (95% confidence interval (CI_95_) = 1.06 (1.03, 1.09); P-value = 1.5×10^−4^, per allele). In conclusion, our study suggests that some genetic loci implicated in determining human lung function also influence cIMT and susceptibility to CAD. The present results should help elucidate the molecular underpinnings of the co-morbidity observed across COPD and CAD.

## Introduction

Chronic obstructive pulmonary disease (COPD) is a condition characterised by impaired airflow to the lungs that worsens over time [1]. The primary risk factor for COPD is long-term exposure to noxious particles and gases, in particular from cigarette smoking, which has been shown to trigger inflammation and abnormal immune responses in the small airways [2]. Local inflammation in the lung may, in turn, trigger systemic inflammatory reactions, such as production of acute-phase proteins in the liver, with potential adverse consequences for non-respiratory organs [3]. The incidence proportion of COPD of any severity grade in smokers reported by observational studies ranges between 15%–40%. The corresponding rates in non-smokers are 8%–15% [4], [5]. As not all smokers contract COPD, it is believed that susceptibility to COPD is highly variable between individuals, and that some of the variability may be explained by genetics, environment and lifestyle, and interactions between these factors [6].

In pulmonary function testing with spirometry, a reduced postbronchodilator FEV_1_/FVC ratio indicates the presence of airflow limitation and is required for the diagnosis of COPD. To study the genetic component of COPD, genome-wide association (GWA) studies have attempted to identify genetic determinants of human lung function in healthy subjects, using spirometry data on Forced Expiratory Volume in one second (FEV)_1_ and its ratio to Forced Vital Capacity (FVC) (FEV_1_/FVC). To date, a total of 26 genetic loci for human lung function have been identified, some of which also seem to be associated with COPD susceptibility, such as the loci at *TNS1*, *RARB*, *FAM13A*, *GSTCD*, *HHIP*, *ADAM19*, *HTR4*, *AGER*, *GPR126*, *C10orf11* and *THSD4* [7], [8], [9], [10], [11].

Multiple studies have reported that cardiovascular disease (CVD), including coronary artery disease (CAD), congestive heart failure, stroke and peripheral arterial disease, is a major contributor to mortality and morbidity in COPD. A recent meta-analysis sought to quantify the CVD risk in COPD using literature data, and observed a 2–5 fold increased CVD risk in patients with COPD compared with age- and sex-matched controls without COPD [12]. The difference persisted after adjustment for known risk factors. Amongst several possible explanations for the strong co-morbidity is that COPD and CAD not only progress in parallel, but also share some common etiologically relevant biological pathways, involving e.g. oxidative stress, matrix remodelling and innate and adaptive immune responses. In the present study, we sought to address this hypothesis by testing genetic loci for spirometric measures as determinants for carotid intima-media thickness (cIMT) and susceptibility to CAD.

## Methods

### SNP selection

Single nucleotide polymorphisms (SNPs) attaining genome-wide significance in four recent GWA studies for either FEV_1_ or the ratio of FEV_1_ to FVC [13], [14], [15], [16] were selected for cross-reference analysis with CAD susceptibility and cIMT. In particular, we selected 26 lead SNPs, representing 26 loci robustly associated with spirometry measures, through a literature search (Table 1).

**Table 1:** Information of the 26 lung function-associated SNPs selected in the present study [13], [14], [15], [16].

			LITERATURE							IMPROVE				
SNP ID	Chr	closest gene	A1	A1 freq	Measure reported	Beta	SE	P	proxy_LD	SNP	A1	A2	A1 freq	n
rs2284746	1	*MFAP2*	G	0.52	FEV1/FVC	–0.040	0.005	7.5×10^−16^	0.87	rs6657613	T	A	0.47	3378
rs993925	1	*TGFB2*	T	0.31	FEV1/FVC	0.034	0.006	1.16×10^−8^	1.00	rs993925	G	A	0.29	3276
rs2571445	2	*TNS1*	G	0.60	FEV1	0.035	0.005	1.11×10^−12^	1.00	rs2571445	A	G	0.40	3378
rs12477314	2	*HDAC4*	T	0.20	FEV1/FVC	0.041	0.006	1.68×10^−12^	1.00	rs12477314	G	A	0.22	3255
rs1529672	3	*RARB*	C	0.83	FEV1/FVC	–0.048	0.006	3.97×10^−14^	1.00	rs1529672	C	A	0.18	3281
rs1344555	3	*MECOM*	T	0.21	FEV1	–0.034	0.006	2.65×10^−8^	1.00	rs1344555	G	A	0.20	3266
rs2869967	4	*FAM13A*	T	0.61	FEV1/FVC	0.035	0.007	1.91×10^−7^	1.00	rs2869967	C	T	0.39	3226
rs10516526	4	*GSTCD*	G	0.06	FEV1	0.089	0.009	2.18×10^−23^	1.00	rs10516526	G	A	0.06	3203
rs12504628	4	*HHIP*	T	0.56	FEV1/FVC	–0.077	0.011	6.48×10^−13^	0.97	rs13147758	G	A	0.45	3378
rs2277027	5	*ADAM19*	A	0.71	FEV1/FVC	0.045	0.007	9.93×10^−11^	1.00	rs2277027	C	A	0.36	3235
rs3995090	5	*HTR4*	C	0.40	FEV1	0.038	0.006	4.29×10^−09^	1.00	rs3995090	C	A	0.43	3283
rs153916	5	*SPATA9*	T	0.55	FEV1/FVC	–0.031	0.005	2.012×10^−8^	1.00	rs153916	A	G	0.44	3292
rs3817928	6	*GPR126*	A	0.78	FEV1/FVC	–0.050	0.008	1.17×10^−09^	1.00	rs11155242	C	A	0.19	3378
rs2070600	6	*AGER*	T	0.05	FEV1/FVC	0.088	0.011	3.07×10^−15^	1.00	rs2070600	T	C	0.04	3370
rs2857595	6	*NCR3*	G	0.81	FEV1/FVC	0.037	0.006	2.28×10^−10^	1.00	rs2857595	A	G	0.17	3378
rs6903823	6	*ZKSCAN3/ZNF323*	G	0.25	FEV1/FVC	−0.021	0.007	1.19×10^−3^	0.95	rs6912584	C	T	0.17	3378
rs2798641	6	*ARMC2*	T	0.18	FEV1/FVC	–0.041	0.007	8.35×10^−9^	1.00	rs2768551	A	G	0.18	3378
rs16909898	9	*PTCH1*	A	0.90	FEV1/FVC	0.059	0.012	5.34×10^−07^	0.85	rs16909981	C	G	0.12	3378
rs7068966	10	*CDC123*	T	0.52	FEV1/FVC	0.033	0.005	6.13×10^−13^	1.00	rs7068966	A	G	0.48	3249
rs7068966	10	*CDC123*	T	0.52	FEV1	0.029	0.004	2.82×10^−12^	1.00	rs7068966	A	G	0.48	3249
rs11001819	10	*C10orf11*	G	0.52	FEV1	–0.029	0.004	2.98×10^−12^	1.00	rs11001819	C	T	0.44	3312
rs11172113	12	*LRP1*	T	0.61	FEV1/FVC	–0.032	0.006	1.24×10^−8^	1.00	rs11172113	C	T	0.38	3377
rs1036429	12	*CCDC38*	T	0.20	FEV1/FVC	0.038	0.006	2.3×10^−11^	1.00	rs1036429	T	C	0.19	3378
rs12899618	15	*THSD4*	G	0.85	FEV1/FVC	0.060	0.008	7.24×10^−15^	0.92	rs7172592	T	C	0.18	3378
rs2865531	16	*CFDP1*	T	0.42	FEV1/FVC	0.031	0.005	1.77×10^−11^	0.93	rs4888378	A	G	0.43	3378
rs12447804	16	*MMP15*	T	0.21	FEV1/FVC	–0.038	0.007	3.59×10^−8^	1.00	rs12447804	G	A	0.21	3282
rs9978142	21	*KCNE2*	T	0.16	FEV1/FVC	−0.043	0.008	2.65×10^−8^	0.91	rs973754	G	A	0.14	3378

Columns 1 to 9 refer to frequencies, beta and p values for the association of SNPs with lung function fenotypes as found in the literature. Columns 10 to 15 refer to the frequencies and total sample sizes of the same or proxy-SNPs that were looked up in IMPROVE.

SNP ID: rs number for the SNPs selected from literature. Chr: chromosome, A1: coded allele, A1 freq: frequency of the coded allele, Measure reported: phenotype for which the SNP reached genome-wide significant association, SE: standard error, P: p-value for association with measure reported, proxy LD: linkage disequilibrium between SNP ID from literature (column 1) and the proxy used for replication in IMPROVE (column 11), proxy SNP: rs number for the proxy SNPs used for replication in IMPROVE, n: number of individuals tested in IMPROVE.

### Association with cIMT measures

The database and biobank of a large, multicenter, European prospective cohort study (acronym: IMPROVE (Carotid **I**ntima **M**edia Thickness (IMT) and IMT-**PR**ogression as Predictors of **V**ascular **E**vents in a High-Risk European Population) was used for studying SNP associations with various cIMT measures. The IMPROVE study was set up for the study of cIMT measures as predictors of incident coronary events, and enrolled 3,711 subjects with at least three independent CAD risk factors. Detailed descriptions of IMPROVE, including the protocols for carotid ultrasound measures have been reported [17], [18]. In the present study, a total of 3,378 subjects were available for the genetic association analyses, which included the mean and maximum IMT of a common carotid segment excluding the first cm proximal to the bifurcation (CC-IMT_mean_ and CC-IMT_max_), mean and maximum IMT in the internal carotid arteries (ICA-IMT_mean_ and ICA-IMT_max_), and the mean and maximum IMT of the bifurcation (Bif-IMT_mean_ and Bif-IMT_max_). Composite IMT variables considering the whole carotid tree, derived from the segment-specific measurements (IMT_mean_, IMT_max_, and IMT_mean-max_ (the average of IMT maxima recorded at the different segments)) were also tested for association.

Six of the SNPs had previously been genotyped on the Illumina CardioMetabochip array. The CardioMetabochip interrogates ≈200,000 SNPs located in regions identified by previous GWA studies of metabolic and cardiovascular traits and diseases. For eight of the lead SNPs, we selected proxy SNPs (r^2^≥0.85) that were present on the CardioMetabochip array. Proxies were selected using SNAP software [19] using 1000 genomes pilot 1 CEU samples as reference. The remaining 12 SNPs were genotyped with TaqMan probes from Applied Biosystems. Quality control procedures for the CardioMetabochip array in IMPROVE have been described [20].

We performed linear regression analyses between the 26 lung function-associated SNPs and different cIMT measures using PLINK (v1.07) [21], assuming an additive genetic model and adjusting for age, gender, body-mass index and the first 3 multidimensional scaling (MDS) dimensions to account for population stratification (based on CardioMetabochip genotype data, see details in [20]). All cIMT variables were logarithmically transformed before statistical analysis because of skewed distributions. All P-values were Bonferroni-corrected (statistical significance set at a P-value≤0.00192).

### Replication

Replication of the rs3995090 association with cIMT measures was pursued in the Rotterdam Study (RSI and RSII) and in the Malmö Diet and Cancer Cohort (MDCC). A description of the samples used for all analyses is included in Section S1. Only measures of CC-IMT_mean_ were available in all replication cohorts. In addition, CC-IMT_max_ measures were available for RSI and RSII. Results from the three replication cohorts were meta-analyzed by using an inverse-variance model with fixed effects as implemented in METAL [22]. Statistical significance for this SNP was set at a P-value≤0.05.

### Association with CAD

We also sought association *in silico* of the 26 lung function SNPs with CAD in 5,775 CAD cases and 7,265 controls using GWA data from the PROCARDIS [23] and Wellcome Trust Case Control Consortium (WTCCC) collections [24]. Association was tested by logistic regression analysis assuming an additive model and adjusting for age, gender, and country using STATA version 11 ( StataCorp LP, College Station, TX, USA). Since PROCARDIS contains related individuals (see Section S1), relatedness was taken into account by setting families as clusters. All P-values were Bonferroni-corrected (statistical significance set at a P-value≤0.00192).

### Association with Gene Expression

SNP rs3995090 was further analyzed, first with respect to its association with expression levels of *HTR4,* and then in relation to the level of expression of adjacent genes (located within ±500 kilobases (kb) of *HTR4*) in a secondary extended search, using data from the Advanced Study of Aortic Pathology (ASAP) and Biobank of Karolinska Endarterectomies (BiKE) data sets [25]. In the ASAP study, mRNA was extracted from biopsies of ascending thoracic aorta intima-media (n = 138), aortic adventitia (n = 133), mammary artery (n = 89), heart (n = 127), and liver (n = 211) from patients undergoing aortic valve surgery. In the BiKE study, RNA was extracted from human plaque tissue (n = 126) and peripheral blood mononuclear cells (n = 96) from patients referred for surgical treatment of severe carotid artery stenosis. Associations between SNP genotype and gene expression level were examined using additive linear models. Rs3995090 was genotyped in both studies with the Illumina 610w-Quad BeadArray.

### Genetic Risk Scores

We calculated weighted and unweighted genetic risk scores (GRS) based on the significant SNPs from the FEV_1_/FVC and FEV_1_ GWAs in the literature and used it as a continuous predictor in logistic/linear regression models with CAD and cIMT-related phenotypes. Unweighted GRS were built considering the number of risk alleles, while weighted GRS were built considering the number of risk alleles weighting them for the beta values reported in literature. Specifically, the GRS for FEV_1_/FVC was built on the following SNPs and beta values (in brackets) derived from [13], [14], [15], [16]: rs153916 (0.031), rs2277027 (0.045), rs12447804 (0.038), rs2857595 (0.037), rs2070600 (0.088), rs2869967 (0.035), rs11172113 (0.032), rs12477314 (0.041), rs1690989 (0.059), rs3817928 (0.05), rs2865531 (0.031), rs7068966 (0.033), rs2284746 (0.04), rs9978142 (0.043), rs993925 (0.034), rs1036429 (0.037), rs12899618 (0.06), rs1529672 (0.048), rs12504628 (0.077) and rs2798641 (0.041). The GRS for FEV_1_ was built on the following SNPs and beta values: rs2571445 (0.035), rs6903823 (0.037), rs10516526 (0.089), rs3995090 (0.038), rs11001819 (0.029), rs1344555 (0.034) and rs7068966 (0.029). Figure S1 shows the frequencies of the number of risk alleles used to calculate unweighted GRS within PROCARDIS and IMPROVE cohorts. Since weighted GRS result from the product of the number of risk alleles and their effect size, the resulting units are arbitrary. For the sake of clarity, weighted GRS were divided in intervals representing total number of possible risk alleles to be comparable to the “increased OR per risk allele” that was calculated for the unweighted scores.

## Results

### Associations with cIMT-related measures

We tested the association between the 26 selected SNPs (or good proxies) and the different cIMT-associated phenotypes. After adjustment for age, gender and the first three MDS, a SNP located in the *HTR4* gene (rs3995090) and a proxy for rs2865531 (located in *CFDP1*) were found to be consistently associated with several of the cIMT-associated phenotypes (Table 2, Table S1). The strongest associations were observed with IMT_mean_ (rs3995090) and IMT_mean-max_ (rs2865531), both composite cIMT variables considering the whole carotid tree and derived from the segment-specific measurements. There was very little change in association after further adjustment for smoking (pack-years) (data not shown). Results stratified by smoking-status are shown in Tables S2–S3.

**Table 2:** SNPs showing significant associations with different IMT measurements.

			CC-IMTmean		CC-IMTmax		ICA-IMTmean		ICA-IMTmax		Bif-IMTmean		Bif_IMTmax		IMTmean		IMTmax		IMTmean-max	
SNP	A1		beta	P	beta	P	beta	P	beta	P	beta	P	beta	P	beta	P	beta	P	beta	P
rs3995090	C		0.003	0.088	0.002	0.380	0.010	0.004	0.012	0.007	0.010	0.002	0.010	0.017	0.007	2.63E-04	0.010	0.010	0.007	0.002
rs4888378	A		–0.005	0.009	–0.007	0.010	–0.011	0.001	–0.016	4.41E-04	–0.013	6.14E-05	–0.018	8.74E-06	–0.009	3.93E-06	–0.019	5.10E-07	–0.010	2.87E-06

Chr: chromosome, A1: coded allele, P: p-value for association with IMT, CC-IMT_mean_: average IMT of the common carotid in a segment excluding the first cm proximal to the bifurcation, CC-IMT_max_: maximum IMT of the common carotid in a segment excluding the first cm proximal to the bifurcation, ICA-IMT_mean_: average IMT of the internal carotid, ICA-IMT_max_: maximum IMT of the internal carotid, Bif-IMT_mean_: average IMT of the bifurcation, Bif-IMT_max_: maximum IMT of the bifurcation, IMT_mean_: average IMT composite value considering the whole carotid tree derived from the segment-specific measurements, IMT_max_: Maximum IMT measure considering the whole carotid tree derived from the segment-specific measurements, IMT_mean-max_: average of the IMT_max_ values for the whole carotid tree derived from the segment-specific measurements. rs4888378 was used as proxy SNP for rs2865531.

A regional look-up to assess the association between other SNPs located in the *HTR4* gene (rs10077690, rs17720191, rs11168048, rs10061244, rs13359903, rs2278392, rs1422636, rs4336354, rs1833710, rs7700268 and rs888961) did not uncover any other significant cIMT association within this gene.

Associations were also investigated under a model where all established CAD risk factors were included, using a stepwise model in SPSS (using log-transformed IMT_mean_ as phenotype). Altogether, systolic blood pressure, diastolic blood pressure, waist-hip ratio, triglycerides, HDL-cholesterol, and LDL-cholesterol explained 7.5% of the variance in this cIMT phenotype, after adjusting for MDS1–3, age and sex. After adjustment for all these covariates, rs3995090 and rs2865531 remained significantly associated with the cIMT phenotypes (Table S4).

GRS-based analyses using the significant SNPs from the FEV_1_/FVC and FEV_1_ GWAs in literature were not significant for association with cIMT phenotypes (Table S5).

### Association with CAD

The minor allele of the SNP located in the *CFDP1* gene (rs2865531T) was associated with a lower risk of CAD (OR(CI_95_) = 0.85(0.79–0.92); P-value = 5.36×10^−5^). The minor allele of the SNP located in *KCNE2* (rs9978142T) was associated with increased risk of CAD (OR(CI_95_) = 1.22 (1.10, 1.35); P-value = 1.23×10^−4^). In addition, the GRS assessing the global effect of all the 7 FEV_1_–robustly associated SNPs from the 4 previous GWAs in literature showed a moderate effect but significant association with CAD risk, OR(CI_95_) for weighted score = 1.05 (1.02, 1.08); P-value = 0.002; OR(CI_95_) for unweighted score = 1.06 (1.03, 1.09); P-value = 1.5×10^−4^ per allele).

The GRS assessing the global effect of the 20 FEV_1_/FVC-robustly associated SNPs from the 4 previous GWAs in literature did not prove to be significantly associated with CAD. Association results for all SNPs is shown in Table S6.

### Replication

Among the two spirometry SNPs that showed significant associations with cIMT measures, rs2865531 has been previously reported as a determinant of cIMT and CAD risk [20]. Likewise, the associations between rs9978142 and rs2865533 and CAD susceptibility were previously established in a large case-control study of CAD [26]; hence, replication was not pursued.

Therefore, we concentrated further replication efforts on SNP rs3995090. Replication of rs3995090 was sought in a total of 12,803 individuals with CC-IMT_mean_ and in 6,679 individuals with CC-IMT_max_ measures. The rs3995090A allele was associated with increased CC-IMT_max_ (beta = 0.006, P-value = 0.044).

### Association with gene expression

Expression levels of *HTR4* in the heart and vessel wall tissues were lower than average (below the 30% percentile of all genes). In peripheral blood mononuclear cells and carotid plaque, the gene was expressed at the 60% percentile of all genes. SNP rs3995090 was not associated with mRNA expression levels of *HTR4* in any of the tissues tested in the ASAP and BiKE studies, although a trend was observed in aortic adventitia at P = 0.0826. In a further expanded search including other neighbouring genes (±500 Kb), rs3995090 was not associated with mRNA levels of other neighbouring genes, after multiple-testing correction for 7 genes in 7 data sets (Table S7).

## Discussion

COPD is the fourth largest cause of death worldwide [27]. Co-morbidities between COPD and other common complex diseases such as CAD may suggest that shared genetic and/or environmental risk factors exist. Several epidemiologic studies have suggested before that CAD is a major contributor to mortality and morbidity in COPD, and that the association between COPD measures and CAD goes beyond the fact that both diseases share common environmental risk factors, such as poor diet, sedentary lifestyle and smoking (reviewed in [28]). Although these studies cannot demonstrate a causal relationship between COPD and CAD, strong evidence suggests that the increased systemic inflammation and oxidative stress associated with COPD contribute to the increased risk of cardiovascular events, and it is plausible that multiple other still unknown pathophysiologic pathways may contribute to the development of both diseases (reviewed in [29]).

In order to explore potential common genetic variants influencing risk of both COPD and cardiovascular disease, we tested 26 SNPs with robust association with human lung function for association with CAD. Since cIMT is considered a robust biomarker for early atherosclerosis, we also tested these 26 lung function-associated SNPs with different measures of cIMT. Of note, inverse relationships between pulmonary function measures adjusted for other risk factors and cIMT have been found in several studies [30], [31], [32], indicating that cIMT may be a robust biomarker for determining cardiovascular morbidity and mortality in COPD [29].

In agreement with our hypothesis that common genetic factors exist between the COPD and CAD, we found two lung function-associated SNPs (rs2865531, located in the *CFDP1* gene and rs9978142 located in the *KCNE2* gene) that were also associated with CAD, the minor allele being associated with lower (rs2865531T) risk and increased risk of CAD (rs9978142T), respectively. In addition, the latter, along with SNP rs3995090 located in the *HTR4* gene, showed strong associations with several cIMT measures. Finally, a GRS, assessing the global effect of all the 7 FEV_1_–associated SNPs from the literature, showed an association with CAD risk. In all, these results indicate that common genetic pathways may exist between COPD and cIMT and CAD, and these are probably independent from the most classical associated factors, such as systolic blood pressure, diastolic blood pressure, waist-hip ratio, triglycerides, HDL-cholesterol, and LDL-cholesterol, since further adjustment for these covariates did not alter the associations found in the present study.

Among the SNPs associated with both diseases, the SNP located in *KNCE2* (rs9982601, proxy for rs973754 (r^2^ = 0.81)) has previously been associated with early-onset myocardial infarction (MI) in a GWA study of 2,967 cases and 3,075 matched controls (OR(CI_95%_) = 1.19 (1.13, 1.27), P = 2×10^−9^) [26]. *KNCE2,* located on chromosome 21, codes for a potassium voltage-gated channel, and mutations in this gene cause inherited arrhythmias [33]. The rare allele of the SNP located in *CFDP1* was recently found to be associated with higher cIMT measures in a gene-centric meta-analysis [20]. Interestingly, this SNP was not associated with expression levels of *CFDP1*, although a strong association was found between rs4888378 alleles and expression levels of a nearby gene (*BCAR1*), which has been implicated in cellular adhesion, migration and proliferation/survival of smooth muscle cells [20], [34], [35].

Our results for rs3995090, located in the *HTR4* region, do not provide solid evidence of an association with a specific gene. The SNP is located in *HTR4*, which is a member of the family of serotonin receptors. However, expression analyses showed that there are no allelic-specific differences in the expression of this gene by rs3995090 genotype. Other mechanisms might be present that explain the effect of rs3995090 in *HTR4*, possibly involving changes at a protein level. Further studies are needed to elucidate the role of this SNP.

To the best of our knowledge, this is the first comprehensive look-up of human lung function robustly-associated loci for association with CAD and cIMT. Although several epidemiologic studies have suggested shared pathophysiologic pathways between both diseases, the present study clearly demonstrates that some human lung function-associated loci are also associated with CAD and cIMT. While further functional studies are warranted to elucidate the role of these genes in the pathophysiology of COPD and CAD, the overall findings made in this and previous studies suggest that there are some shared genetic pathways involved in airway obstruction and cardiovascular risk. This notion opens new interesting perspectives in understanding the co-morbidity of two important, common complex diseases.

## Supporting Information

Figure S1 **Frequencies of the FEV1 and FEV1/FCV number of risk alleles in IMPROVE and PROCARDIS.**
(PDF)

Table S1 **Association between all lung function-associated SNPs from 4 GWA studies in the literature and IMT phenotypes in IMPROVE.**
(DOCX)

Table S2 **Association between all lung function-associated SNPs from 4 GWA studies in the literature and IMT phenotypes in smokers from IMPROVE.**
(DOCX)

Table S3 **Association between all lung function-associated SNPs from 4 GWA studies in the literature and IMT phenotypes in non-smokers from IMPROVE.**
(DOCX)

Table S4 **Association between all lung function-associated SNPs from 4 GWA studies in the literature and IMT phenotypes in IMPROVE after adjusting for age, sex, MDS1–3, systolic blood pressure, diastolic blood pressure, waist-hip ratio, tryglicerides, HDL-cholesterol, and LDL-cholesterol.**
(DOCX)

Table S5 **Association between weighted Genetic Risk Scores (GRS) and IMT phenotypes in IMPROVE, after adjustment for age, sex and the three first multidimensional scaling (MDS) dimensions.**
(DOCX)

Table S6 **Association between all lung function-associated SNPs from 4 GWA studies in the literature and CAD risk in PROCARDIS+WTCCC (5,775 CAD cases and 7,265 controls).**
(DOCX)

Table S7 **Association between rs3995090 and HTR4 expression levels in different tissues.**
(DOCX)

Section S1 **Sample descriptions.**
(DOCX)
