# Supplementary material for: Common Genetic Determinants of Lung Function, Subclinical Atherosclerosis and Risk of Coronary Artery Disease
Source: PLoS One. 2014 Aug 5;9(8):e104082. doi: 10.1371/journal.pone.0104082 (PMC4122436; doi:10.1371/journal.pone.0104082)
Supplement: Figure S1 — Frequencies of the FEV1 and FEV1/FCV number of risk alleles in IMPROVE and PROCARDIS. (PDF) [file pone.0104082.s001.pdf]

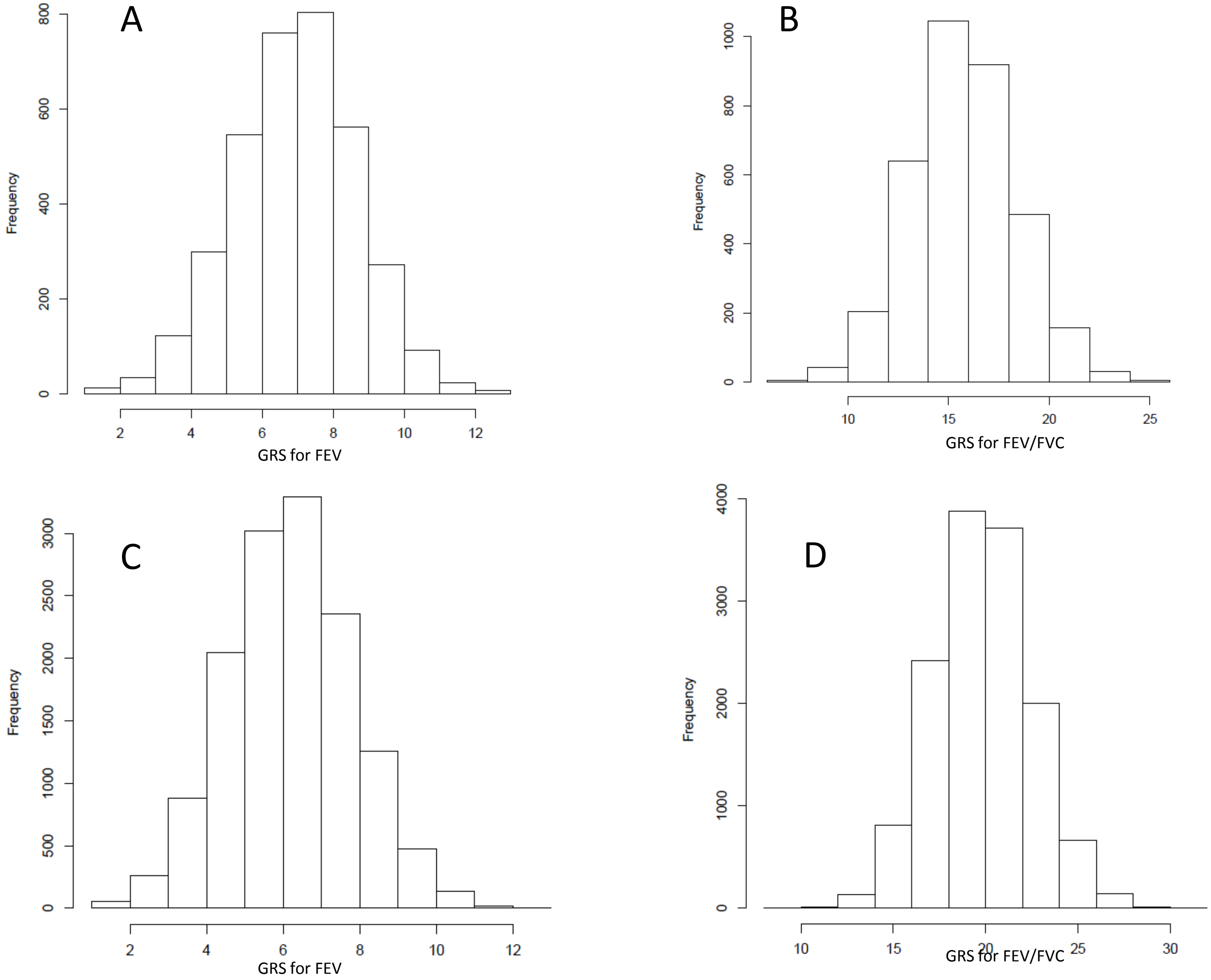

Figure S1: A and B show frequencies of the FEV1 and FEV1/FCV number of risk alleles respectively in IMPROVE. C and D show frequencies of the FEV1 and FEV1/FCV number of risk alleles respectively in PROCARDIS.
