# Supplementary material for: Common Genetic Determinants of Lung Function, Subclinical Atherosclerosis and Risk of Coronary Artery Disease
Source: PLoS One. 2014 Aug 5;9(8):e104082. doi: 10.1371/journal.pone.0104082 (PMC4122436; doi:10.1371/journal.pone.0104082)
Supplement: Table S1 — Association between all lung function-associated SNPs from 4 GWA studies in the literature and IMT phenotypes in IMPROVE. (DOCX) [file pone.0104082.s002.docx]

Table S1: Association between all lung function-associated SNPs from 4 GWA studies in the literature and IMT phenotypes in IMPROVE (N=3,442).

|  |  | CC-IMTmean | | CC-IMTmax | | ICA-IMTmean | | ICA-IMTmax | | Bif-IMTmean | | Bif-IMTmax | | IMTmean |  | IMTmax |  | IMTmean-max | |
| --- | --- | --- | --- | --- | --- | --- | --- | --- | --- | --- | --- | --- | --- | --- | --- | --- | --- | --- | --- |
| SNP | A1 | beta | P | beta | P | beta | P | beta | P | beta | P | beta | P | BETA | P | beta | P | beta | P |
| rs6657613 | T | -0.001 | 0.724 | -0.002 | 0.418 | -3.33E-04 | 0.921 | -0.004 | 0.378 | -0.005 | 0.092 | -0.008 | 0.036 | -0.002 | 0.220 | -0.009 | 0.019 | -0.004 | 0.072 |
| rs993925 | G | 0.002 | 0.282 | 0.004 | 0.223 | -0.004 | 0.338 | -0.002 | 0.675 | -0.004 | 0.250 | -0.004 | 0.328 | -0.001 | 0.509 | -0.003 | 0.418 | -0.001 | 0.695 |
| rs2571445 | A | -0.001 | 0.650 | -0.002 | 0.466 | 0.001 | 0.849 | 0.001 | 0.908 | 0.002 | 0.488 | 0.002 | 0.537 | 0.001 | 0.785 | 0.001 | 0.781 | 0.001 | 0.691 |
| rs12477314 | G | -1.95E-04 | 0.926 | -0.001 | 0.683 | -0.004 | 0.343 | -0.006 | 0.287 | 0.001 | 0.774 | 0.001 | 0.839 | -0.001 | 0.826 | -0.001 | 0.767 | -0.001 | 0.779 |
| rs1529672 | C | 0.001 | 0.775 | 0.004 | 0.288 | -0.001 | 0.754 | -0.005 | 0.426 | 0.003 | 0.519 | 0.004 | 0.408 | 2.46E-04 | 0.926 | 0.001 | 0.777 | -0.001 | 0.831 |
| rs1344555 | G | 0.001 | 0.595 | 0.002 | 0.662 | -0.003 | 0.535 | -0.003 | 0.624 | -0.003 | 0.515 | -3.79E-04 | 0.939 | -3.19E-04 | 0.901 | -4.21E-05 | 0.993 | 0.001 | 0.838 |
| rs2869967 | C | -3.58E-04 | 0.839 | -3.47E-04 | 0.902 | 0.001 | 0.784 | 3.23E-04 | 0.944 | 0.005 | 0.154 | 0.007 | 0.065 | 0.002 | 0.325 | 0.007 | 0.070 | 0.002 | 0.268 |
| rs10516526 | G | -2.61E-04 | 0.943 | -0.004 | 0.538 | -0.002 | 0.742 | -0.009 | 0.341 | 0.001 | 0.833 | 6.60E-05 | 0.994 | 0.001 | 0.879 | -0.007 | 0.357 | -0.002 | 0.657 |
| rs17035960 | T | 0.003 | 0.468 | 0.002 | 0.721 | 2.75E-04 | 0.970 | 0.003 | 0.764 | -3.16E-05 | 0.996 | -0.003 | 0.731 | 4.42E-04 | 0.919 | 0.004 | 0.671 | 4.80E-04 | 0.918 |
| rs13147758 | G | 0.001 | 0.763 | 0.001 | 0.763 | 2.88E-04 | 0.932 | 0.002 | 0.702 | -0.005 | 0.128 | -0.007 | 0.055 | -0.001 | 0.612 | -0.005 | 0.193 | -0.001 | 0.734 |
| rs153916 | A | 0.001 | 0.681 | 0.001 | 0.633 | -0.003 | 0.386 | -0.004 | 0.380 | -0.002 | 0.648 | -0.003 | 0.456 | -0.001 | 0.570 | -0.003 | 0.447 | -0.002 | 0.346 |
| rs12374521 | C | 0.002 | 0.268 | 0.002 | 0.393 | 0.005 | 0.139 | 0.005 | 0.235 | 0.004 | 0.281 | 0.004 | 0.301 | 0.003 | 0.116 | 0.004 | 0.335 | 0.003 | 0.151 |
| rs3995090 | C | 0.003 | 0.079 | 0.003 | 0.361 | 0.010 | 0.004 | 0.012 | 0.007 | 0.010 | 0.002 | 0.010 | 0.016 | 0.007 | 2.54E-04 | 0.010 | 0.010 | 0.007 | 0.002 |
| rs2277027 | C | -0.002 | 0.307 | 1.35E-04 | 0.962 | 0.001 | 0.792 | 9.58E-05 | 0.983 | -0.001 | 0.710 | 3.75E-04 | 0.927 | -0.001 | 0.615 | 2.45E-06 | 1.000 | -9.42E-05 | 0.966 |
| rs2857595 | A | 0.002 | 0.421 | 0.007 | 0.076 | -0.002 | 0.591 | -0.003 | 0.598 | -0.002 | 0.647 | -0.001 | 0.801 | -0.001 | 0.790 | -0.001 | 0.888 | 0.001 | 0.725 |
| rs6912584 | C | 0.003 | 0.146 | 0.003 | 0.389 | 0.005 | 0.239 | 0.005 | 0.417 | 0.009 | 0.029 | 0.011 | 0.029 | 0.006 | 0.030 | 0.006 | 0.223 | 0.006 | 0.040 |
| rs2070600 | T | -0.002 | 0.619 | -0.004 | 0.526 | 0.001 | 0.950 | -0.003 | 0.816 | 0.008 | 0.286 | 0.003 | 0.749 | 0.003 | 0.545 | 0.005 | 0.579 | -0.001 | 0.887 |
| rs2768551 | A | 3.69E-04 | 0.864 | 0.001 | 0.688 | 0.005 | 0.276 | 0.008 | 0.148 | 0.002 | 0.568 | 0.001 | 0.767 | 0.002 | 0.404 | 0.003 | 0.513 | 0.003 | 0.258 |
| rs11155242 | C | -0.001 | 0.686 | -0.001 | 0.790 | -0.001 | 0.833 | -0.003 | 0.593 | -0.004 | 0.340 | -0.005 | 0.334 | -0.001 | 0.625 | -0.003 | 0.525 | -0.001 | 0.586 |
| rs16909981 | C | 0.001 | 0.713 | 0.005 | 0.247 | -0.002 | 0.633 | -0.001 | 0.879 | 0.001 | 0.885 | 3.92E-04 | 0.946 | 9.93E-05 | 0.974 | 0.001 | 0.804 | 0.002 | 0.581 |
| rs7068966 | A | -0.002 | 0.280 | -0.001 | 0.815 | 9.44E-05 | 0.978 | 9.57E-05 | 0.983 | 0.002 | 0.447 | 0.004 | 0.258 | 0.001 | 0.774 | 0.005 | 0.195 | 0.001 | 0.505 |
| rs11001819 | C | 0.001 | 0.672 | -7.65E-05 | 0.978 | 0.001 | 0.834 | -0.003 | 0.502 | -0.004 | 0.180 | -0.005 | 0.179 | 0.000 | 0.808 | -0.005 | 0.200 | -0.001 | 0.677 |
| rs11172113 | C | -4.65E-04 | 0.788 | -0.003 | 0.235 | 0.002 | 0.556 | 0.001 | 0.779 | -0.008 | 0.011 | -0.008 | 0.056 | -0.003 | 0.211 | -0.007 | 0.062 | -0.003 | 0.227 |
| rs1036429 | T | 1.80E-04 | 0.935 | 0.001 | 0.851 | 0.002 | 0.577 | 0.002 | 0.669 | -0.004 | 0.307 | -0.002 | 0.720 | -0.001 | 0.785 | -0.001 | 0.880 | -1.20E-04 | 0.965 |
| rs7172592 | T | 0.003 | 0.233 | -0.002 | 0.642 | 0.002 | 0.630 | -4.67E-04 | 0.935 | -3.20E-04 | 0.940 | -0.004 | 0.411 | 0.002 | 0.530 | -0.002 | 0.747 | 2.66E-04 | 0.923 |
| rs12447804 | G | -0.002 | 0.253 | -0.003 | 0.431 | -0.004 | 0.325 | -0.004 | 0.445 | -0.002 | 0.654 | -4.71E-04 | 0.922 | -0.002 | 0.357 | -0.002 | 0.668 | -0.001 | 0.609 |
| rs4888378 | A | -0.004 | 0.010 | -0.007 | 0.010 | -0.011 | 0.001 | -0.016 | 4.41E-04 | -0.013 | 4.84E-05 | -0.018 | 6.12E-06 | -0.009 | 3.56E-06 | -0.019 | 3.98E-07 | -0.010 | 2.33E-06 |
| rs973754 | G | 0.001 | 0.547 | -2.87E-04 | 0.941 | -4.82E-04 | 0.920 | 0.002 | 0.723 | -0.002 | 0.680 | -0.001 | 0.821 | -0.001 | 0.847 | 0.003 | 0.588 | -4.73E-04 | 0.876 |

A1: coded allele, P: p-value for association with IMT, CC-IMT_mean_: average IMT of the common carotid in a segment excluding the first cm proximal to the bifurcation, CC-IMT_max_: maximum IMT of the common carotid in a segment excluding the first cm proximal to the bifurcation, ICA-IMT_mean_: average IMT of the internal carotid, ICA-IMT_max_: maximum IMT of the internal carotid, Bif-IMT_mean_: average IMT of the bifurcation, Bif-IMT_max_: maximum IMT of the bifurcation, IMT_mean_: average IMT composite value considering the whole carotid tree derived from the segment-specific measurements, IMT_max_: Maximum IMT measure considering the whole carotid tree derived from the segment-specific measurements, IMT_mean-max_: average of the IMT_max_ values for the whole carotid tree derived from the segment-specific measurements.
