# Supplementary material for: Common Genetic Determinants of Lung Function, Subclinical Atherosclerosis and Risk of Coronary Artery Disease
Source: PLoS One. 2014 Aug 5;9(8):e104082. doi: 10.1371/journal.pone.0104082 (PMC4122436; doi:10.1371/journal.pone.0104082)
Supplement: Table S2 — Association between all lung function-associated SNPs from 4 GWA studies in the literature and IMT phenotypes in smokers from IMPROVE. (DOCX) [file pone.0104082.s003.docx]

Table S2: Association between all lung function-associated SNPs from 4 GWA studies in the literature and IMT phenotypes in smokers from IMPROVE (N=515).

|  |  | CC-IMTmean | | CC-IMTmax | | ICA-IMTmean | | ICA-IMTmax | | Bif-IMTmean | | Bif-IMTmax | | IMTmean |  | IMTmax |  | IMTmean-max | |
| --- | --- | --- | --- | --- | --- | --- | --- | --- | --- | --- | --- | --- | --- | --- | --- | --- | --- | --- | --- |
| SNP | A1 | beta | P | beta | P | beta | P | beta | P | beta | P | beta | P | beta | P | beta | P | beta | P |
| rs6657613 | T | 0.002 | 0.685 | -0.004 | 0.615 | 0.003 | 0.711 | 0.001 | 0.911 | -0.007 | 0.376 | -0.009 | 0.389 | -0.002 | 0.729 | -0.008 | 0.415 | -0.003 | 0.649 |
| rs993925 | G | 0.005 | 0.379 | 0.006 | 0.517 | -2.87E-04 | 0.979 | -0.001 | 0.922 | -0.010 | 0.317 | -0.006 | 0.590 | -0.002 | 0.769 | -0.009 | 0.414 | -4.71E-04 | 0.943 |
| rs2571445 | A | 0.004 | 0.370 | 0.007 | 0.354 | 0.009 | 0.326 | 0.011 | 0.388 | 0.003 | 0.720 | 0.005 | 0.600 | 0.004 | 0.414 | 0.011 | 0.265 | 0.007 | 0.209 |
| rs12477314 | G | -2.71E-04 | 0.964 | -0.006 | 0.541 | -0.012 | 0.338 | -0.031 | 0.052 | -0.002 | 0.839 | 0.006 | 0.649 | -0.004 | 0.519 | -0.008 | 0.496 | -0.010 | 0.191 |
| rs1529672 | C | 0.015 | 0.013 | 0.019 | 0.051 | 0.022 | 0.068 | 0.022 | 0.158 | 0.014 | 0.199 | 0.013 | 0.301 | 0.016 | 0.016 | 0.016 | 0.201 | 0.016 | 0.024 |
| rs1344555 | G | -0.009 | 0.155 | -0.008 | 0.437 | -0.006 | 0.613 | -0.015 | 0.365 | -0.005 | 0.656 | -1.62E-04 | 0.990 | -0.006 | 0.393 | -0.006 | 0.642 | -0.006 | 0.431 |
| rs2869967 | C | 1.98E-04 | 0.968 | 0.003 | 0.735 | 0.010 | 0.326 | 0.014 | 0.287 | 0.011 | 0.223 | 0.015 | 0.167 | 0.007 | 0.207 | 0.018 | 0.083 | 0.009 | 0.137 |
| rs10516526 | G | -0.010 | 0.368 | -0.022 | 0.199 | 0.005 | 0.816 | -0.003 | 0.920 | 0.007 | 0.726 | -0.006 | 0.801 | 0.002 | 0.838 | -0.009 | 0.675 | -0.003 | 0.820 |
| rs17035960 | T | 0.020 | 0.073 | 0.032 | 0.065 | -0.004 | 0.847 | 0.018 | 0.533 | 0.007 | 0.702 | 0.002 | 0.918 | 0.010 | 0.409 | 0.025 | 0.265 | 0.016 | 0.236 |
| rs13147758 | G | 1.17E-04 | 0.980 | -0.004 | 0.627 | -0.011 | 0.254 | -0.012 | 0.338 | -0.013 | 0.105 | -0.016 | 0.107 | -0.008 | 0.148 | -0.014 | 0.149 | -0.008 | 0.183 |
| rs153916 | A | 0.001 | 0.831 | 0.008 | 0.305 | -0.002 | 0.860 | 0.001 | 0.946 | -0.004 | 0.679 | -0.007 | 0.508 | -0.001 | 0.819 | -0.006 | 0.517 | 0.001 | 0.871 |
| rs12374521 | C | 0.008 | 0.110 | 0.013 | 0.103 | -0.001 | 0.936 | 0.002 | 0.884 | 0.003 | 0.712 | -0.006 | 0.546 | 0.005 | 0.369 | -0.002 | 0.807 | 0.006 | 0.339 |
| rs3995090 | C | 0.001 | 0.813 | 2.60E-04 | 0.974 | 0.003 | 0.776 | 0.008 | 0.542 | 0.006 | 0.484 | -3.96E-04 | 0.970 | 0.004 | 0.441 | 0.003 | 0.793 | 0.005 | 0.460 |
| rs2277027 | C | -0.003 | 0.603 | 0.005 | 0.499 | -0.005 | 0.612 | -0.007 | 0.548 | 0.008 | 0.325 | 0.014 | 0.178 | 2.26E-04 | 0.966 | 0.003 | 0.730 | 0.002 | 0.731 |
| rs2857595 | A | 1.35E-04 | 0.984 | 0.003 | 0.798 | -0.002 | 0.892 | -0.001 | 0.939 | 0.004 | 0.722 | 0.006 | 0.692 | 0.001 | 0.908 | -0.006 | 0.670 | 0.005 | 0.525 |
| rs6912584 | C | -0.008 | 0.220 | -0.009 | 0.433 | 0.005 | 0.740 | 0.008 | 0.667 | 0.015 | 0.207 | 0.027 | 0.064 | 0.002 | 0.839 | 0.004 | 0.793 | 0.007 | 0.388 |
| rs2070600 | T | 0.007 | 0.553 | -0.004 | 0.823 | 0.013 | 0.585 | 0.020 | 0.520 | 0.005 | 0.819 | 0.007 | 0.788 | 0.008 | 0.540 | 0.015 | 0.527 | 0.006 | 0.661 |
| rs2768551 | A | 0.006 | 0.341 | 0.007 | 0.457 | 0.006 | 0.634 | 0.012 | 0.471 | 0.008 | 0.481 | 0.015 | 0.271 | 0.005 | 0.468 | 0.012 | 0.339 | 0.007 | 0.388 |
| rs11155242 | C | 0.003 | 0.660 | 0.000 | 0.989 | -0.007 | 0.564 | -0.018 | 0.223 | -0.026 | 0.010 | -0.027 | 0.031 | -0.009 | 0.146 | -0.021 | 0.079 | -0.010 | 0.156 |
| rs16909981 | C | 0.008 | 0.251 | 0.018 | 0.105 | 0.001 | 0.923 | 0.010 | 0.576 | -0.004 | 0.743 | 3.70E-04 | 0.980 | 0.004 | 0.575 | 0.011 | 0.445 | 0.010 | 0.220 |
| rs7068966 | A | -0.004 | 0.420 | 0.002 | 0.763 | -0.004 | 0.695 | -0.008 | 0.492 | 0.008 | 0.335 | 0.013 | 0.183 | 0.001 | 0.826 | 0.006 | 0.517 | 0.004 | 0.536 |
| rs11001819 | C | 0.005 | 0.234 | 1.30E-04 | 0.986 | 0.007 | 0.469 | -0.003 | 0.817 | -0.001 | 0.925 | -0.010 | 0.322 | 0.003 | 0.588 | -0.010 | 0.287 | 0.001 | 0.849 |
| rs11172113 | C | 0.003 | 0.525 | 0.002 | 0.777 | -0.005 | 0.615 | -0.010 | 0.417 | -0.001 | 0.947 | 0.006 | 0.552 | -0.001 | 0.909 | 0.001 | 0.945 | 0.001 | 0.882 |
| rs1036429 | T | -1.64E-04 | 0.979 | 0.005 | 0.608 | -0.006 | 0.649 | -0.011 | 0.499 | 0.014 | 0.216 | 0.018 | 0.196 | 0.002 | 0.791 | 0.003 | 0.815 | 0.002 | 0.831 |
| rs7172592 | T | -0.004 | 0.576 | -0.010 | 0.360 | -0.003 | 0.799 | -0.015 | 0.393 | -0.001 | 0.918 | -0.008 | 0.570 | -0.003 | 0.660 | -0.002 | 0.884 | -0.007 | 0.373 |
| rs12447804 | G | 0.002 | 0.690 | -0.004 | 0.719 | -0.016 | 0.198 | -0.009 | 0.568 | 0.005 | 0.648 | 0.009 | 0.526 | 1.44E-04 | 0.984 | 0.004 | 0.757 | 0.002 | 0.757 |
| rs4888378 | A | -0.004 | 0.421 | -0.007 | 0.367 | -0.018 | 0.072 | -0.023 | 0.086 | -0.007 | 0.412 | -0.013 | 0.229 | -0.009 | 0.105 | -0.021 | 0.042 | -0.009 | 0.123 |
| rs973754 | G | 0.004 | 0.513 | -2.21E-04 | 0.984 | -0.015 | 0.258 | -0.010 | 0.556 | -0.020 | 0.099 | -0.026 | 0.077 | -0.009 | 0.256 | -0.023 | 0.105 | -0.009 | 0.264 |

A1: coded allele, P: p-value for association with IMT, CC-IMT_mean_: average IMT of the common carotid in a segment excluding the first cm proximal to the bifurcation, CC-IMT_max_: maximum IMT of the common carotid in a segment excluding the first cm proximal to the bifurcation, ICA-IMT_mean_: average IMT of the internal carotid, ICA-IMT_max_: maximum IMT of the internal carotid, Bif-IMT_mean_: average IMT of the bifurcation, Bif-IMT_max_: maximum IMT of the bifurcation, IMT_mean_: average IMT composite value considering the whole carotid tree derived from the segment-specific measurements, IMT_max_: Maximum IMT measure considering the whole carotid tree derived from the segment-specific measurements, IMT_mean-max_: average of the IMT_max_ values for the whole carotid tree derived from the segment-specific measurements.
