# Supplementary material for: Common Genetic Determinants of Lung Function, Subclinical Atherosclerosis and Risk of Coronary Artery Disease
Source: PLoS One. 2014 Aug 5;9(8):e104082. doi: 10.1371/journal.pone.0104082 (PMC4122436; doi:10.1371/journal.pone.0104082)
Supplement: Table S3 — Association between all lung function-associated SNPs from 4 GWA studies in the literature and IMT phenotypes in non-smokers from IMPROVE. (DOCX) [file pone.0104082.s004.docx]

Table S3: Association between all lung function-associated SNPs from 4 GWA studies in the literature and IMT phenotypes in non-smokers from IMPROVE (N=2925).

|  |  | CC-IMTmean | | CC-IMTmax | | ICA-IMTmean | | ICA-IMTmax | | Bif-IMTmean | | Bif-IMTmax | | IMTmean |  | IMTmax |  | IMTmean-max | |
| --- | --- | --- | --- | --- | --- | --- | --- | --- | --- | --- | --- | --- | --- | --- | --- | --- | --- | --- | --- |
| SNP | A1 | beta | P | beta | P | beta | P | beta | P | beta | P | beta | P | BETA | P | beta | P | beta | P |
| rs6657613 | T | -0.001 | 0.566 | -0.002 | 0.530 | -0.001 | 0.878 | -0.004 | 0.366 | -0.005 | 0.166 | -0.008 | 0.066 | -0.002 | 0.274 | -0.008 | 0.037 | -0.004 | 0.093 |
| rs993925 | G | 0.002 | 0.342 | 0.004 | 0.221 | -0.004 | 0.331 | -0.002 | 0.742 | -0.003 | 0.460 | -0.004 | 0.444 | -0.001 | 0.646 | -0.002 | 0.641 | -0.001 | 0.803 |
| rs2571445 | A | -0.002 | 0.367 | -0.004 | 0.218 | -0.001 | 0.846 | -0.001 | 0.836 | 0.002 | 0.490 | 0.002 | 0.594 | 3.84E-05 | 0.986 | -2.95E-04 | 0.942 | -1.02E-04 | 0.964 |
| rs12477314 | G | 1.82E-05 | 0.993 | -2.24E-04 | 0.949 | -0.002 | 0.571 | -0.001 | 0.813 | 0.002 | 0.628 | 0.001 | 0.912 | 3.52E-04 | 0.893 | 1.07E-04 | 0.983 | 0.001 | 0.702 |
| rs1529672 | C | -0.002 | 0.357 | 0.001 | 0.843 | -0.006 | 0.206 | -0.010 | 0.104 | 3.39E-04 | 0.942 | 0.002 | 0.710 | -0.003 | 0.292 | -0.002 | 0.756 | -0.004 | 0.168 |
| rs1344555 | G | 0.003 | 0.199 | 0.003 | 0.365 | -0.002 | 0.735 | 0.000 | 0.971 | -0.002 | 0.601 | -1.21E-04 | 0.982 | 0.001 | 0.747 | 0.002 | 0.737 | 0.002 | 0.497 |
| rs2869967 | C | -2.03E-04 | 0.914 | -4.91E-04 | 0.870 | -0.001 | 0.861 | -0.002 | 0.684 | 0.004 | 0.299 | 0.006 | 0.158 | 0.001 | 0.565 | 0.005 | 0.211 | 0.001 | 0.554 |
| rs10516526 | G | 0.002 | 0.678 | -1.34E-04 | 0.983 | -0.002 | 0.764 | -0.008 | 0.422 | 0.003 | 0.713 | 0.003 | 0.708 | 0.002 | 0.741 | -0.005 | 0.568 | -0.001 | 0.907 |
| rs17035960 | T | 4.73E-04 | 0.904 | -0.002 | 0.756 | 0.002 | 0.820 | 0.002 | 0.860 | -0.001 | 0.937 | -0.003 | 0.739 | -4.86E-04 | 0.917 | 0.001 | 0.885 | -0.001 | 0.795 |
| rs13147758 | G | 3.47E-04 | 0.849 | 0.001 | 0.640 | 0.002 | 0.527 | 0.004 | 0.408 | -0.004 | 0.274 | -0.006 | 0.130 | -9.33E-05 | 0.966 | -0.004 | 0.355 | 2.12E-04 | 0.926 |
| rs153916 | A | 0.001 | 0.713 | 1.35E-04 | 0.963 | -0.003 | 0.370 | -0.005 | 0.324 | -0.001 | 0.836 | -0.002 | 0.675 | -0.001 | 0.640 | -0.002 | 0.632 | -0.002 | 0.304 |
| rs12374521 | C | 0.001 | 0.620 | 0.001 | 0.829 | 0.006 | 0.090 | 0.006 | 0.206 | 0.004 | 0.289 | 0.006 | 0.155 | 0.003 | 0.170 | 0.005 | 0.237 | 0.003 | 0.229 |
| rs3995090 | C | 0.003 | 0.107 | 0.002 | 0.420 | 0.010 | 0.004 | 0.012 | 0.012 | 0.010 | 0.003 | 0.011 | 0.012 | 0.008 | 0.001 | 0.010 | 0.013 | 0.007 | 0.004 |
| rs2277027 | C | -0.002 | 0.275 | -0.001 | 0.626 | 0.001 | 0.705 | 0.001 | 0.907 | -0.004 | 0.318 | -0.003 | 0.514 | -0.002 | 0.419 | -0.001 | 0.734 | -0.001 | 0.651 |
| rs2857595 | A | 0.002 | 0.431 | 0.007 | 0.079 | -0.003 | 0.562 | -0.004 | 0.545 | -0.003 | 0.500 | -0.003 | 0.633 | -0.001 | 0.681 | -1.83E-04 | 0.974 | 1.42E-04 | 0.964 |
| rs6912584 | C | 0.005 | 0.031 | 0.005 | 0.184 | 0.006 | 0.186 | 0.005 | 0.399 | 0.010 | 0.038 | 0.010 | 0.069 | 0.007 | 0.013 | 0.008 | 0.153 | 0.006 | 0.038 |
| rs2070600 | T | -0.003 | 0.437 | -0.004 | 0.572 | -0.001 | 0.868 | -0.006 | 0.614 | 0.009 | 0.310 | 0.002 | 0.837 | 0.002 | 0.697 | 0.003 | 0.730 | -0.002 | 0.715 |
| rs2768551 | A | -0.001 | 0.827 | 3.76E-04 | 0.918 | 0.005 | 0.300 | 0.008 | 0.181 | 0.002 | 0.730 | -4.96E-04 | 0.925 | 0.002 | 0.522 | 0.002 | 0.688 | 0.003 | 0.366 |
| rs11155242 | C | -0.002 | 0.472 | -0.001 | 0.748 | 1.15E-04 | 0.980 | -4.81E-04 | 0.935 | -9.50E-05 | 0.983 | -0.001 | 0.844 | 0.000 | 0.969 | -2.29E-04 | 0.964 | -7.17E-05 | 0.980 |
| rs16909981 | C | -1.93E-04 | 0.944 | 0.002 | 0.567 | -0.003 | 0.574 | -0.003 | 0.659 | 0.002 | 0.765 | 3.69E-04 | 0.953 | -0.001 | 0.850 | -4.08E-04 | 0.947 | 1.98E-04 | 0.954 |
| rs7068966 | A | -0.001 | 0.458 | -0.001 | 0.756 | 0.001 | 0.814 | 0.002 | 0.736 | 0.001 | 0.676 | 0.003 | 0.523 | 0.001 | 0.802 | 0.005 | 0.270 | 0.001 | 0.624 |
| rs11001819 | C | -2.65E-04 | 0.885 | -4.08E-04 | 0.889 | -0.001 | 0.880 | -0.003 | 0.494 | -0.005 | 0.149 | -0.004 | 0.295 | -0.001 | 0.576 | -0.004 | 0.333 | -0.001 | 0.536 |
| rs11172113 | C | -0.001 | 0.724 | -0.004 | 0.229 | 0.004 | 0.241 | 0.005 | 0.302 | -0.009 | 0.013 | -0.009 | 0.036 | -0.002 | 0.331 | -0.007 | 0.088 | -0.002 | 0.303 |
| rs1036429 | T | 2.05E-04 | 0.930 | -8.69E-05 | 0.981 | 0.004 | 0.391 | 0.005 | 0.428 | -0.008 | 0.091 | -0.005 | 0.319 | -0.001 | 0.661 | -0.001 | 0.776 | -0.001 | 0.852 |
| rs7172592 | T | 0.004 | 0.076 | 3.18E-04 | 0.932 | 0.004 | 0.386 | 0.003 | 0.582 | 0.001 | 0.828 | -0.002 | 0.694 | 0.003 | 0.235 | 3.69E-04 | 0.944 | 0.002 | 0.424 |
| rs12447804 | G | -0.003 | 0.193 | -0.002 | 0.536 | -0.002 | 0.643 | -0.003 | 0.608 | -0.003 | 0.540 | -0.002 | 0.766 | -0.002 | 0.359 | -0.002 | 0.624 | -0.002 | 0.549 |
| rs4888378 | A | -0.004 | 0.016 | -0.007 | 0.017 | -0.010 | 0.005 | -0.015 | 0.002 | -0.014 | 4.51E-05 | -0.019 | 9.69E-06 | -0.009 | 1.32E-05 | -0.019 | 2.88E-06 | -0.010 | 7.08E-06 |
| rs973754 | G | 0.001 | 0.740 | -4.15E-04 | 0.919 | 0.002 | 0.725 | 0.004 | 0.545 | 0.001 | 0.845 | 0.003 | 0.646 | 0.001 | 0.826 | 0.007 | 0.232 | 0.001 | 0.790 |

A1: coded allele, P: p-value for association with IMT, CC-IMT_mean_: average IMT of the common carotid in a segment excluding the first cm proximal to the bifurcation, CC-IMT_max_: maximum IMT of the common carotid in a segment excluding the first cm proximal to the bifurcation, ICA-IMT_mean_: average IMT of the internal carotid, ICA-IMT_max_: maximum IMT of the internal carotid, Bif-IMT_mean_: average IMT of the bifurcation, Bif-IMT_max_: maximum IMT of the bifurcation, IMT_mean_: average IMT composite value considering the whole carotid tree derived from the segment-specific measurements, IMT_max_: Maximum IMT measure considering the whole carotid tree derived from the segment-specific measurements, IMT_mean-max_: average of the IMT_max_ values for the whole carotid tree derived from the segment-specific measurements.
