# Supplementary material for: Common Genetic Determinants of Lung Function, Subclinical Atherosclerosis and Risk of Coronary Artery Disease
Source: PLoS One. 2014 Aug 5;9(8):e104082. doi: 10.1371/journal.pone.0104082 (PMC4122436; doi:10.1371/journal.pone.0104082)
Supplement: Table S4 — Association between all lung function-associated SNPs from 4 GWA studies in the literature and IMT phenotypes in IMPROVE after adjusting for age, sex, MDS1–3, systolic blood pressure, diastolic blood pressure, waist-hip ratio, tryglicerides, HDL-cholesterol, and LDL-cholesterol. (DOCX) [file pone.0104082.s005.docx]

Table S4: Association between all lung function-associated SNPs from 4 GWA studies in the literature and IMT phenotypes in IMPROVE after adjusting for age, sex, MDS1-3, systolic blood pressure, diastolic blood pressure, waist-hip ratio, tryglicerides, HDL-cholesterol, and LDL-cholesterol (N=3,442).

|  |  | CC-IMTmean | | CC-IMTmax | | ICA-IMTmean | | ICA-IMTmax | | Bif-IMTmean | | Bif-IMTmax | | IMTmean |  | IMTmax |  | IMTmean-max | |
| --- | --- | --- | --- | --- | --- | --- | --- | --- | --- | --- | --- | --- | --- | --- | --- | --- | --- | --- | --- |
| SNP | A1 | beta | P | beta | P | beta | P | beta | P | beta | P | beta | P | BETA | P | beta | P | beta | P |
| rs6657613 | T | -0.001 | 0.740 | -0.002 | 0.431 | -2.35E-04 | 0.943 | -0.004 | 0.400 | -0.005 | 0.094 | -0.008 | 0.038 | -0.002 | 0.228 | -0.008 | 0.022 | -0.004 | 0.078 |
| rs993925 | G | 0.002 | 0.256 | 0.004 | 0.203 | -0.004 | 0.318 | -0.002 | 0.662 | -0.004 | 0.238 | -0.004 | 0.329 | -0.002 | 0.477 | -0.003 | 0.404 | -0.001 | 0.689 |
| rs2571445 | A | -0.001 | 0.389 | -0.003 | 0.280 | -9.60E-05 | 0.977 | -3.43E-04 | 0.938 | 0.002 | 0.616 | 0.002 | 0.654 | -1.55E-04 | 0.937 | 2.72E-04 | 0.942 | 1.35E-04 | 0.948 |
| rs12477314 | G | 1.58E-04 | 0.938 | -0.001 | 0.751 | -0.003 | 0.442 | -0.005 | 0.336 | 0.002 | 0.691 | 0.001 | 0.815 | -8.07E-05 | 0.973 | -0.001 | 0.804 | -4.70E-04 | 0.854 |
| rs1529672 | C | 1.68E-04 | 0.939 | 0.003 | 0.357 | -0.002 | 0.639 | -0.005 | 0.352 | 0.002 | 0.640 | 0.004 | 0.485 | -4.68E-04 | 0.856 | 4.86E-04 | 0.922 | -0.001 | 0.658 |
| rs1344555 | G | 0.001 | 0.476 | 0.002 | 0.542 | -0.002 | 0.637 | -0.002 | 0.714 | -0.002 | 0.604 | 7.20E-05 | 0.988 | 2.12E-04 | 0.932 | 4.79E-04 | 0.920 | 0.001 | 0.693 |
| rs2869967 | C | -0.001 | 0.444 | -0.002 | 0.566 | -1.94E-04 | 0.955 | -0.001 | 0.876 | 0.004 | 0.206 | 0.007 | 0.077 | 0.001 | 0.577 | 0.007 | 0.092 | 0.002 | 0.449 |
| rs10516526 | G | 8.94E-05 | 0.980 | -0.003 | 0.594 | -0.002 | 0.743 | -0.009 | 0.335 | 0.001 | 0.875 | -2.28E-04 | 0.978 | 0.001 | 0.882 | -0.008 | 0.329 | -0.002 | 0.665 |
| rs17035960 | T | 0.001 | 0.711 | 0.001 | 0.900 | -0.002 | 0.795 | 0.001 | 0.899 | -0.001 | 0.843 | -0.004 | 0.674 | -0.001 | 0.770 | 0.002 | 0.763 | -0.001 | 0.887 |
| rs13147758 | G | 4.65E-04 | 0.780 | 0.001 | 0.746 | -1.50E-04 | 0.964 | 0.001 | 0.783 | -0.005 | 0.116 | -0.008 | 0.051 | -0.001 | 0.546 | -0.005 | 0.163 | -0.001 | 0.693 |
| rs153916 | A | 0.001 | 0.499 | 0.002 | 0.483 | -0.002 | 0.492 | -0.003 | 0.465 | -0.001 | 0.717 | -0.003 | 0.505 | -0.001 | 0.722 | -0.002 | 0.513 | -0.002 | 0.456 |
| rs12374521 | C | 0.002 | 0.272 | 0.002 | 0.408 | 0.005 | 0.144 | 0.005 | 0.252 | 0.003 | 0.290 | 0.004 | 0.312 | 0.003 | 0.119 | 0.003 | 0.365 | 0.003 | 0.163 |
| rs3995090 | C | 0.003 | 0.075 | 0.002 | 0.361 | 0.010 | 0.004 | 0.012 | 0.006 | 0.010 | 0.002 | 0.010 | 0.012 | 0.007 | 1.89E-04 | 0.010 | 0.007 | 0.007 | 0.001 |
| rs2277027 | C | -0.001 | 0.523 | 0.001 | 0.682 | 0.002 | 0.611 | 0.001 | 0.814 | -0.001 | 0.871 | 0.001 | 0.787 | -2.58E-04 | 0.900 | 0.001 | 0.808 | 0.001 | 0.747 |
| rs2857595 | A | 0.001 | 0.703 | 0.005 | 0.150 | -0.004 | 0.433 | -0.004 | 0.476 | -0.003 | 0.567 | -0.002 | 0.741 | -0.002 | 0.546 | -0.001 | 0.780 | 1.52E-04 | 0.958 |
| rs6912584 | C | 0.003 | 0.156 | 0.003 | 0.427 | 0.005 | 0.242 | 0.005 | 0.420 | 0.009 | 0.029 | 0.011 | 0.029 | 0.006 | 0.030 | 0.006 | 0.222 | 0.006 | 0.041 |
| rs2070600 | T | -0.001 | 0.822 | -0.003 | 0.647 | 0.002 | 0.791 | -0.001 | 0.909 | 0.009 | 0.236 | 0.003 | 0.716 | 0.004 | 0.382 | 0.006 | 0.520 | 1.10E-04 | 0.983 |
| rs2768551 | A | 6.29E-06 | 0.998 | 0.001 | 0.788 | 0.004 | 0.318 | 0.008 | 0.165 | 0.002 | 0.639 | 0.001 | 0.819 | 0.002 | 0.491 | 0.003 | 0.555 | 0.003 | 0.306 |
| rs11155242 | C | -0.002 | 0.406 | -0.002 | 0.545 | -0.002 | 0.582 | -0.005 | 0.404 | -0.005 | 0.212 | -0.006 | 0.232 | -0.002 | 0.330 | -0.004 | 0.338 | -0.003 | 0.332 |
| rs16909981 | C | 4.78E-04 | 0.847 | 0.004 | 0.301 | -0.003 | 0.532 | -0.002 | 0.801 | -1.85E-04 | 0.969 | -2.96E-04 | 0.959 | -0.001 | 0.855 | 0.001 | 0.880 | 0.001 | 0.683 |
| rs7068966 | A | -0.002 | 0.236 | -0.001 | 0.771 | -3.72E-04 | 0.911 | -3.54E-04 | 0.936 | 0.002 | 0.529 | 0.004 | 0.287 | 2.43E-04 | 0.901 | 0.005 | 0.227 | 0.001 | 0.570 |
| rs11001819 | C | 3.17E-04 | 0.849 | -0.001 | 0.789 | 1.02E-04 | 0.976 | -0.004 | 0.386 | -0.005 | 0.106 | -0.006 | 0.117 | -0.001 | 0.552 | -0.006 | 0.119 | -0.002 | 0.455 |
| rs11172113 | C | -0.001 | 0.752 | -0.003 | 0.210 | 0.002 | 0.582 | 0.001 | 0.794 | -0.009 | 0.008 | -0.008 | 0.050 | -0.003 | 0.172 | -0.007 | 0.057 | -0.003 | 0.200 |
| rs1036429 | T | -0.001 | 0.747 | -0.001 | 0.879 | 0.001 | 0.782 | 0.001 | 0.850 | -0.005 | 0.208 | -0.003 | 0.593 | -0.002 | 0.492 | -0.002 | 0.698 | -0.001 | 0.678 |
| rs7172592 | T | 0.003 | 0.245 | -0.002 | 0.599 | 0.002 | 0.695 | -0.001 | 0.858 | -0.001 | 0.808 | -0.005 | 0.343 | 0.001 | 0.629 | -0.002 | 0.635 | -7.68E-05 | 0.977 |
| rs12447804 | G | -0.002 | 0.353 | -0.002 | 0.546 | -0.004 | 0.397 | -0.004 | 0.521 | -0.001 | 0.747 | 9.17E-05 | 0.985 | -0.002 | 0.469 | -0.001 | 0.768 | -0.001 | 0.753 |
| rs4888378 | A | -0.004 | 0.028 | -0.006 | 0.022 | -0.010 | 0.002 | -0.015 | 0.001 | -0.013 | 9.15E-05 | -0.017 | 1.10E-05 | -0.009 | 1.10E-05 | -0.019 | 8.08E-07 | -0.010 | 6.80E-06 |
| rs973754 | G | 0.002 | 0.499 | -1.86E-04 | 0.961 | -2.92E-04 | 0.951 | 0.002 | 0.695 | -0.002 | 0.682 | -0.001 | 0.830 | -4.32E-04 | 0.876 | 0.003 | 0.566 | -3.54E-04 | 0.905 |

A1: coded allele, P: p-value for association with IMT, CC-IMT_mean_: average IMT of the common carotid in a segment excluding the first cm proximal to the bifurcation, CC-IMT_max_: maximum IMT of the common carotid in a segment excluding the first cm proximal to the bifurcation, ICA-IMT_mean_: average IMT of the internal carotid, ICA-IMT_max_: maximum IMT of the internal carotid, Bif-IMT_mean_: average IMT of the bifurcation, Bif-IMT_max_: maximum IMT of the bifurcation, IMT_mean_: average IMT composite value considering the whole carotid tree derived from the segment-specific measurements, IMT_max_: Maximum IMT measure considering the whole carotid tree derived from the segment-specific measurements, IMT_mean-max_: average of the IMT_max_ values for the whole carotid tree derived from the segment-specific measurements.
