# Supplementary material for: Common Genetic Determinants of Lung Function, Subclinical Atherosclerosis and Risk of Coronary Artery Disease
Source: PLoS One. 2014 Aug 5;9(8):e104082. doi: 10.1371/journal.pone.0104082 (PMC4122436; doi:10.1371/journal.pone.0104082)
Supplement: Table S5 — Association between weighted Genetic Risk Scores (GRS) and IMT phenotypes in IMPROVE, after adjustment for age, sex and the three first multidimensional scaling (MDS) dimensions. (DOCX) [file pone.0104082.s006.docx]

Table S5: Association between weighted Genetic Risk Scores (GRS) and IMT phenotypes in IMPROVE, after adjustment for age, sex and the three first multidimensional scaling (MDS) dimensions.

|  | FEV/FVC score | |  | FEV score |  |  |
| --- | --- | --- | --- | --- | --- | --- |
|  | beta | SE | p | beta | SE | p |
| CC-IMTmean | 2.86E-04 | 0.0002 | 0.08 | -1.3E-05 | 0.0001 | 0.89 |
| CC-IMTmax | 4.70E-04 | 0.0003 | 0.071 | -4.8E-05 | 0.0001 | 0.749 |
| ICA-IMTmean | 1.36E-04 | 0.0003 | 0.677 | -7.9E-05 | 0.0002 | 0.674 |
| ICA-IMT max | 3.82E-04 | 0.0004 | 0.368 | -6.9E-05 | 0.0002 | 0.778 |
| Bif-IMTmean | 3.00E-04 | 0.0003 | 0.337 | -1.4E-04 | 0.0002 | 0.433 |
| Bif-IMTmax | 2.85E-04 | 0.0004 | 0.446 | -1.7E-04 | 0.0002 | 0.439 |
| IMTmean | 2.31E-04 | 0.0002 | 0.234 | -9.3E-05 | 0.0001 | 0.401 |
| IMTmax | 4.16E-04 | 0.0004 | 0.253 | -1.2E-04 | 0.0002 | 0.566 |
| IMTmean-max | 3.11E-04 | 0.0002 | 0.13 | -7.7E-05 | 0.0001 | 0.514 |
