# Supplementary material for: Common Genetic Determinants of Lung Function, Subclinical Atherosclerosis and Risk of Coronary Artery Disease
Source: PLoS One. 2014 Aug 5;9(8):e104082. doi: 10.1371/journal.pone.0104082 (PMC4122436; doi:10.1371/journal.pone.0104082)
Supplement: Table S6 — Association between all lung function-associated SNPs from 4 GWA studies in the literature and CAD risk in PROCARDIS+WTCCC (5,775 CAD cases and 7,265 controls). (DOCX) [file pone.0104082.s007.docx]

Table S6: Association between all lung function-associated SNPs from 4 GWA studies in the literature and CAD risk in PROCARDIS+WTCCC (5,775 CAD cases and 7,265 controls).

| chr | SNP | A1 | freq | beta | se | p | interval |
| --- | --- | --- | --- | --- | --- | --- | --- |
| 1 | rs2284746 | C | 0.47 | 0.04 | 0.04 | 0.275 | 1.04(0.97,1.13) |
| 1 | rs993925 | C | 0.66 | -0.01 | 0.05 | 0.904 | 0.99(0.9,1.09) |
| 2 | rs2571445 | G | 0.59 | -0.09 | 0.04 | 0.018 | 0.91(0.84,0.98) |
| 2 | rs12477314 | C | 0.79 | -0.01 | 0.05 | 0.909 | 0.99(0.91,1.09) |
| 3 | rs1529672 | C | 0.83 | 0.01 | 0.05 | 0.800 | 1.01(0.92,1.12) |
| 3 | rs1344555 | C | 0.79 | -0.14 | 0.05 | 0.003 | 0.87(0.79,0.95) |
| 4 | rs2869967 | T | 0.60 | -0.02 | 0.04 | 0.562 | 0.98(0.9,1.06) |
| 4 | rs10516526 | A | 0.93 | -0.03 | 0.08 | 0.709 | 0.97(0.84,1.13) |
| 4 | rs12504628 | T | 0.60 | -0.04 | 0.04 | 0.281 | 0.96(0.88,1.04) |
| 5 | rs153916 | C | 0.45 | -0.01 | 0.04 | 0.893 | 0.99(0.92,1.08) |
| 5 | rs3995090 | C | 0.41 | 0.05 | 0.04 | 0.226 | 1.05(0.97,1.13) |
| 5 | rs2277027 | A | 0.65 | 0.02 | 0.04 | 0.667 | 1.02(0.94,1.1) |
| 6 | rs6903823 | A | 0.77 | 0.04 | 0.05 | 0.324 | 1.05(0.96,1.14) |
| 6 | rs2857595 | G | 0.80 | -0.03 | 0.05 | 0.550 | 0.97(0.88,1.07) |
| 6 | rs2070600 | C | 0.94 | -0.12 | 0.08 | 0.122 | 0.89(0.76,1.03) |
| 6 | rs2798641 | C | 0.81 | 0.04 | 0.05 | 0.383 | 1.04(0.95,1.15) |
| 6 | rs3817928 | A | 0.79 | -0.06 | 0.05 | 0.214 | 0.94(0.86,1.03) |
| 9 | rs16909898 | A | 0.91 | 0.09 | 0.07 | 0.227 | 1.09(0.95,1.25) |
| 10 | rs7068966 | C | 0.48 | 0.11 | 0.04 | 0.005 | 1.12(1.03,1.21) |
| 10 | rs11001819 | G | 0.51 | 0.02 | 0.04 | 0.556 | 1.02(0.95,1.11) |
| 12 | rs11172113 | C | 0.41 | -0.04 | 0.04 | 0.320 | 0.96(0.89,1.04) |
| 12 | rs1036429 | C | 0.79 | -0.03 | 0.05 | 0.575 | 0.97(0.89,1.07) |
| 15 | rs12899618 | G | 0.84 | -0.03 | 0.05 | 0.540 | 0.97(0.87,1.08) |
| 16 | rs12447804 | C | 0.78 | -0.05 | 0.05 | 0.261 | 0.95(0.86,1.04) |
| 16 | rs2865531 | T | 0.40 | -0.16 | 0.04 | 5.36E-05 | 0.85(0.79,0.92) |
| 21 | rs9978142 | T | 0.19 | 0.20 | 0.05 | 1.23E-04 | 1.22(1.1,1.35) |
