# Supplementary material for: Common Genetic Determinants of Lung Function, Subclinical Atherosclerosis and Risk of Coronary Artery Disease
Source: PLoS One. 2014 Aug 5;9(8):e104082. doi: 10.1371/journal.pone.0104082 (PMC4122436; doi:10.1371/journal.pone.0104082)
Supplement: Table S7 — Association between rs3995090 and HTR4 expression levels in different tissues. (DOCX) [file pone.0104082.s008.docx]

Table S7: Association between rs3995090 and HTR4 expression levels in different tissues.

|  | Sample size | HTR4 Probeset ID* | Percent genes with lower expression than HTR4† | HTR4 and rs3995090 association P-value‡ |
| --- | --- | --- | --- | --- |
| Mammary artery intima-media | 89 | 2880552 | 31.1% | 0.797 |
| Liver | 212 | 2880552 | 20.1% | 0.568 |
| Aorta intima-media | 139 | 2880552 | 29.3% | 0.875 |
| Aorta adventitia | 133 | 2880552 | 21.6% | 0.0826 |
| Heart | 127 | 2880552 | 22.7% | 0.827 |
| PBMC | 98 | 207578_s_at | 57.4% | 0.549 |
| Carotid Plaque | 127 | 207578_s_at | 57.5% | 0.134 |

* Note that the PBMC and carotid plaque microarray type had three probesets for HTR4. The non-207578_s_at probesets had very low expression values all below 3.7.

† For each gene with more than one probeset, the max value was taken in this calculation

‡For PBMC and carotid plaque, the P-value is from 207578_s_at, which had the strongest P-value of association of three available probesets
