# Supplementary material for: Common Genetic Determinants of Lung Function, Subclinical Atherosclerosis and Risk of Coronary Artery Disease
Source: PLoS One. 2014 Aug 5;9(8):e104082. doi: 10.1371/journal.pone.0104082 (PMC4122436; doi:10.1371/journal.pone.0104082)
Supplement: Section S1 — Sample descriptions. (DOCX) [file pone.0104082.s009.docx]

**Section S1: Sample descriptions**

**Precocious Coronary Artery Disease Study (PROCARDIS) cases and controls**

The PROCARDIS study consists of CAD cases and controls from four European countries: the UK, Italy, Sweden, and Germany. CAD was defined as myocardial infarction (MI), acute coronary syndrome, unstable or stable angina, or need for coronary artery bypass surgery or percutaneous coronary intervention, and was diagnosed before 66 years of age; 80% of cases had a sibling fulfilling the same criteria for CAD. Subjects with self-reported non-European ancestry were excluded. Among the ‘genetically enriched’ CAD cases, 70% had suffered MI. Individuals from PROCARDIS containing genome-wide genotyping data include 2,133 cases who are either a full or half sibling, and 115 Trios. All others individuals are unrelated.

Controls with no personal or sibling history of CAD before the age of 66 years were contemporaneously recruited by use of the same infrastructure. For each of the CAD cases, one control was recruited fulfilling the following criteria: same sex and ethnicity; age within a 5-year interval around the matched case's age; and without a personal or sibling history of CAD before the age of 66 years.

**Wellcome Trust Case Control Consortium (WTCCC) controls**

The WTCCC controls included UK population controls recruited from the National Blood Service population and controls from the 1958 British Birth Cohort, a UK population-based study of individuals born in 1958 who had been genotyped previously for the WTCCC.

The **Rotterdam Study (RS)** is a prospective population-based cohort of individuals aged 55 years or older in the municipality of Rotterdam, the Netherlands. The baseline examination was completed between 1990 and 1993 (RS-I). In 1999, the cohort was extended to include inhabitants who reached the age of 55 years after the baseline examination and persons aged 55 years or older who migrated into the research area (RS-II). The current analysis was based on the data from 4699 participants at the first examination of the original cohort (RS-I) and 1980 participants at the first examination of the extended cohort (RS-II) for whom both genotype data and carotid intima-media thickness measurements were available.

The **Malmö Diet and Cancer (MDC) -cardiovascular (CV) study** is a prospective population-based cohort that consists of 6,103 subjects aged 45-69 years (60% women), living in the city in Malmö, Sweden, of whom 5,540 also accepted invitation for blood sampling under standardized fasting circumstances for the study of the epidemiology of carotid artery disease. Using B-mode ultrasound the right carotid artery was scanned within a predefined window of 3 cm of the distal CCA, the bifurcation and 1 cm of the internal and external carotid artery. IMT was measured "off-line" in the far wall according to the leading edge principle, using a specially designed computer-assisted image analyzing system. In the bulb, the maximal IMT-values were chosen as measurements, whereas in the CCA mean IMT-values of a 1 cm distance just proximal to the bulb were noted. The occurrence of plaques was defined as focal IMT > 1,2 mm.

The **ASAP** biobank consists of a collection of biopsies from the liver, the aortic wall, the internal mammary artery and the myocardium, as well as adipose tissue from the epicardial, intra-abdominal and subcutaneous fat depots, taken from close to 600 patients who have undergone aortic valve surgery in the Department of Thoracic Surgery at the Karolinska University Hospital. Genome-wide high-density SNP-genotyping data (Illumina 610K quad and Illumina 200K iSelect Cardiometabochip) and gene expression profiles (Affymetrix GeneChip Human Exon 1.0 ST arrays) from all target tissue biopsies are available.

The **BiKE** tissue biobank comprises plaques (for immunohistology, lipidomics etcetera) and plaque mRNA, serum/plasma and DNA from >500 patients with carotid stenosis; the complete transcriptome has been explored in >150 plaques (Affymetrix U133Plus2.0 arrays), the donors of which have undergone genotyping on the Illumina 610K BeadArray platform.
